# Enhancing Antibacterial Efficacy: Combining Novel Bacterial Topoisomerase Inhibitors with Efflux Pump Inhibitors and Other Agents Against Gram-Negative Bacteria

**DOI:** 10.3390/antibiotics13111081

**Published:** 2024-11-13

**Authors:** Maša Zorman, Maja Kokot, Irena Zdovc, Lidija Senerovic, Mina Mandic, Nace Zidar, Andrej Emanuel Cotman, Martina Durcik, Lucija Peterlin Mašič, Nikola Minovski, Marko Anderluh, Martina Hrast Rambaher

**Affiliations:** 1 Theory Department, Laboratory for Cheminformatics, National Institute of Chemistry, Hajdrihova 19, 1001 Ljubljana, Slovenia; masa.zorman@ki.si (M.Z.); maja.kokot@sandoz.com (M.K.); nikola.minovski@ki.si (N.M.); 2 Department of Pharmaceutical Chemistry, Faculty of Pharmacy, University of Ljubljana, Aškerčeva Cesta 7, 1000 Ljubljana, Slovenia; nace.zidar@ffa.uni-lj.si (N.Z.); andrej.emanuel.cotman@ffa.uni-lj.si (A.E.C.); martina.durcik@ffa.uni-lj.si (M.D.); lucija.peterlinmasic@ffa.uni-lj.si (L.P.M.); marko.anderluh@ffa.uni-lj.si (M.A.); 3 Institute of Microbiology and Parasitology, Veterinary Faculty, University of Ljubljana, Gerbičeva 60, 1000 Ljubljana, Slovenia; irena.zdovc@vf.uni-lj.si; 4 Laboratory for Microbial Molecular Genetics and Ecology, Institute of Molecular Genetics and Genetic Engineering, University of Belgrade, Vojvode Stepe 444a, 11042 Belgrade, Serbia; lidijasenerovic@imgge.bg.ac.rs (L.S.); mina.mandic@imgge.bg.ac.rs (M.M.)

**Keywords:** topoisomerase inhibitors, NBTIs, efflux pump inhibitors, ESKAPE pathogens, antimicrobial resistance, bacterial biofilms

## Abstract

Background: The novel bacterial topoisomerase inhibitors (NBTIs) developed in our laboratory show potent on-target enzyme inhibition but suffer from low activity against Gram-negative bacteria. Methods: With the aim of improving the antibacterial activity of our compounds against Gram-negative bacteria, we tested them in combination with different efflux pump inhibitors (EPIs), a strategy that showed promise in several other classes of antimicrobials. We also investigated the combined effect of NBTIs with ATP-competitive inhibitors of bacterial type II topoisomerases (ACIs), as well as the antibiofilm properties of our compounds and the combination with EPIs against early and mature *Acietobacter baumannii* biofilm. Results: Our results demonstrate that combinations of NBTIs with EPI Phenylalanine-arginyl-β-naphthylamide significantly reduce the corresponding NBTIs’ minimal inhibitory concentration values and show potentiation of *A. baumannii* biofilm inhibition as compared to NBTIs alone. Although combinations of NBITs and ACIs did not show synergistic effects, the FIC index value calculations revealed additive effects for all the combinations of a selected NBTI in combination with three ACIs in all the assayed Gram-negative bacteria from the ESKAPE group. Conclusions: These results show for the first time that combinations of NBTIs with either EPIs or a different class of the topoisomerase inhibitors may be a beneficial strategy to combat difficult-to-treat bacterial infections.

## 1. Introduction

Antibiotic resistance is one of the greatest threats to global health, with 1.27 million deaths being directly linked to it according to a 2019 estimate [1]. Staggeringly, around three-quarters of antimicrobial-resistance-associated deaths were attributable to only six pathogen species—*Escherichia coli*, *Staphylococcus aureus*, *Klebsiella pneumoniae*, *Streptococcus pneumoniae*, *Acinetobacter baumannii*, and *Pseudomonas aeruginosa*—some of which belong to the multidrug-resistant ESKAPE pathogen group [2,3]. While some bacteria are intrinsically resistant to certain antibiotics due to inherent structural or functional characteristics, such as low bacterial cell wall permeability, constitutive expression of efflux pumps, and biofilm formation, others can acquire resistance to antibiotics due to either horizontal gene transfer, e.g., plasmid-mediated transmission of resistance genes, or genetic mutations that lead to target modifications or modifications of antibiotic-modifying enzymes [4,5,6,7,8]. Gram-negative bacteria are more susceptible to antibiotics compared to Gram-positive ones due to inherent structural differences, such as low permeability due to the presence of an outer membrane and drug efflux via specific efflux pumps [9,10]. Additionally, bacterial biofilms offer bacteria an improved living environment, wherein the resistance seems to depend on multicellular strategies that may differ from the mechanisms of resistance in free-floating (planktonic) bacteria [11].

Multidrug efflux systems are able to pump out various substrates, including several classes of antibiotics [12,13]. The overexpression of a specific efflux pump in bacteria commonly leads to the development of resistance as the intracellular concentration of the antibiotic decreases, making the bacteria less sensitive to the active substance. The minimum inhibitory concentration (MIC) of those strains overexpressing efflux pumps is usually two to eight times greater than that of a susceptible strain of the same species but can also be much greater [14,15,16,17]. The concomitant use of efflux pump inhibitors (EPIs) with already known antibiotics is an interesting approach to improve the clinical efficacy of antibiotics, but this is not clinically validated [18]. EPIs can potentially enhance antibiotic efficacy by reducing the intrinsic bacterial resistance to antibiotics, reversing the acquired resistance associated with efflux pump overexpression, and reducing the frequency of emergence of resistant mutant strains [19,20,21,22].

**Figure 1:**
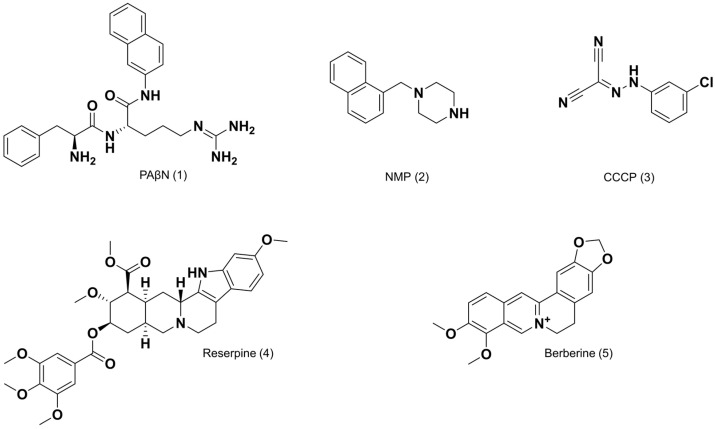
Structural representations of the investigated efflux pump inhibitors.

Phenylalanine-arginyl-β-naphthylamide (PAβN, Figure 1) is a broad-spectrum competitive EPI and the first compound to reduce the intrinsic resistance of *P. aeruginosa* to levofloxacin [23,24,25]. Although PAβN caused a reduction in levofloxacin MIC in the wild-type *P. aeruginosa*, as well as in strains overexpressing efflux pumps, its unfavorable pharmacokinetic and toxicological profiles prevent its clinical use [19,21,22]. 1-Naphthylmethyl-piperazine (NMP, Figure 1) is one of the most potent unsubstituted arylpiperazines, with the ability to increase the intracellular concentration of several antibiotics in *A. baumannii* and several *Enterobacteriaceae* but not in *P. aeruginosa* [26]. In contrast to PAβN, the (low) intrinsic antibacterial activity of NMP and its cellular accumulation are not enhanced in cells with inactivated efflux pumps [27]. Carbonyl cyanide 3-chlorophenylhydrazone (CCCP, Figure 1) is an example of a proton-motive force inhibitor that disrupts the proton-motive force of the membrane, thereby reducing the bacterial cell viability and promoting cell death [28]. Unfortunately, it is highly noxious and cytotoxic. Reserpine (Figure 1), an inhibitor of vesicular monoamine transporters and blocker of transmembrane calcium entry, is routinely used to evaluate the efflux pump activity of Gram-positive bacteria; however, the concentrations necessary to block bacterial efflux are neurotoxic [28]. Berberine (Figure 1), a naturally occurring product, also exhibits activity against efflux pumps; nevertheless, problems with its synthesis, purification, stability, and solubility, as well as its potential toxicity, have reduced the interest in its further development as an EPI [21,29].

Bacteria occur either as free-living cells or as aggregates of cells surrounded by a self-produced polymer matrix that adhere to each other and/or to a surface, called biofilms [30]. Biofilm-growing microorganisms have different specific characteristics compared to planktonic microorganisms, e.g., growth rate and gene transcription, and may exhibit as much as a 1000-fold increase in antibiotic resistance against conventional antibacterials [6,7]. It is speculated that the main driving forces of antimicrobial resistance in biofilms are slowed diffusion of antibacterials into the biofilm matrix, the altered chemical microenvironment within the biofilm, and cell differentiation similar to spore formation [11]. This evidence does not, however, exclude the possibility that conventional resistance mechanisms, such as drug pumps, are expressed in biofilms and contribute to antibiotic resistance in biofilms [11]. Some EPIs have been shown to inhibit biofilm formation in Gram-negative bacteria that showed upregulated expression of efflux pumps [28]. Other mechanisms of action of antibiofilm molecules include inhibition of quorum sensing, inhibition of adhesion, and disruption of various extracellular targets involved in various signaling pathways, all of which may be achieved by a variety of different structural classes of compounds [31].

Bacterial type II topoisomerases are a well-validated class of targets for antibacterial chemotherapy. Although two classes of topoisomerase inhibitors, fluoroquinolones and aminocoumarins, have reached the drug market, aminocoumarins have since been withdrawn due to their ineffectiveness, and fluoroquinolones are increasingly losing efficacy due to emerging resistant bacterial strains. Novel bacterial topoisomerase inhibitors (NBTIs) are a new class of inhibitors that bind to a separate binding site on the enzyme and show no cross-resistance with either fluoroquinolones or aminocoumarins [32,33,34]. ATP-competitive inhibitors of bacterial type II topoisomerases (ACIs), which act at the same site as aminocoumarins, are also being developed and show improved antibacterial properties [35]. Both NBTIs and ACIs exhibit excellent antibacterial activity against Gram-positive bacteria, but the activity of the current inhibitors against Gram-negative bacteria in the planktonic form and biofilms is not optimal due to problems with membrane permeability and efflux from the cell [36,37,38,39,40]. The reasons for their ineffectiveness in biofilms are likely even more complex.

**Figure 2:**
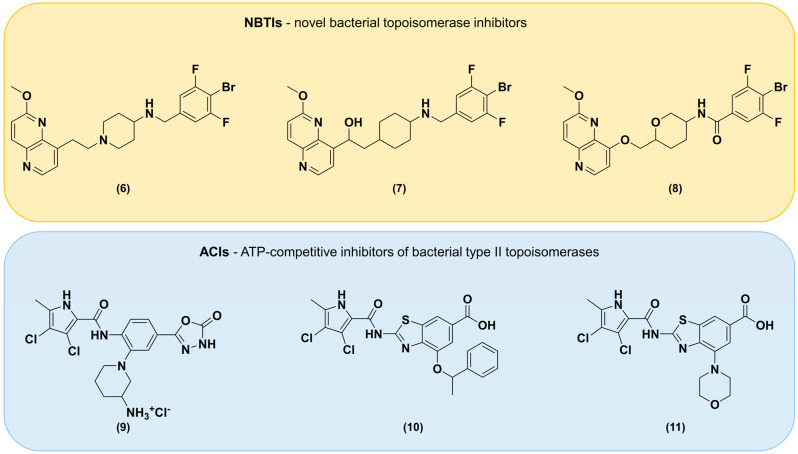
Structural representations of the investigated novel bacterial topoisomerase inhibitors (**6** (*N*-(4-bromo-3,5-difluorobenzyl)-1-(2-(6-methoxy-1,5-naphthyridin-4-yl)ethyl)piperidin-4-amine), **7** (2-(4-((4-bromo-3,5-difluorobenzyl)amino)cyclohexyl)-1-(6-methoxy-1,5-naphthyridin-4-yl)ethan-1-ol), and **8** (4-bromo-3,5-difluoro-*N*-(6-(((6-methoxy-1,5-naphthyridin-4-yl)oxy)methyl)tetrahydro-2*H*-pyran-3-yl)benzamide)) and ATP-competitive inhibitors of bacterial type II topoisomerases (**9** (1-(2-(3,4-dichloro-5-methyl-1*H*-pyrrole-2-carboxamido)-5-(5-oxo-4,5-dihydro-1,3,4-oxadiazol-2-yl)phenyl)piperidin-3-aminium chloride), **10** (2-(3,4-dichloro-5-methyl-1*H*-pyrrole-2-carboxamido)-4-(1-phenylethoxy)benzo[*d*]thiazole-6-carboxylic acid), and **11** (2-(3,4-dichloro-5-methyl-1*H*-pyrrole-2-carboxamido)-4-morpholinobenzo[*d*]thiazole-6-carboxylic acid)).

Combining antimicrobial agents with EPIs is a promising strategy aimed at improving and maintaining the antibacterial potency of antibacterials. This was confirmed to work in other classes of antibacterial agents but never in NBTIs [19,22,41,42]. The coadministration of EPIs and NBTIs is expected to increase the intracellular concentration of NBTIs that are substrates of efflux pumps, decrease the intrinsic bacterial resistance to antibiotics, reverse the acquired resistance associated with efflux pump overexpression, and reduce the frequency of emergence of resistant mutant strains [29]. All these aspects prompted us to further investigate the use of NBTIs in combination with various EPIs in Gram-negative bacteria. Another well-known strategy for combating the ineffectiveness of individual drugs in the treatment of bacterial infections is the coadministration of two or more antibacterials [43]. The main reasons for this are broad-spectrum coverage, treatment of polymicrobial infections, prevention of a high mutation rate of the bacteria due to the use of a single antibiotic, dose reduction and corresponding reduction in dose-related toxicity, and antimicrobial synergistic activity [44]. With the aim of improving the antibacterial activity of our compounds, we have investigated the potential synergistic effect of NBTIs and selected ACIs (Figure 2) in different Gram-negative bacteria. Furthermore, the ability of an antibacterial to inhibit both bacteria found in planktonic form as well as bacteria in biofilms could expand its overall functionality and broaden its use. Since some EPIs are known biofilm disruptors, we investigated the antibiofilm effects of NBTIs alone, as well as NBTIs in combination with EPIs.

## 2. Results

### 2.1. Intrinsic Antibacterial Activity of NBTIs

The intrinsic antibacterial activities of the presented compounds were evaluated against *E. coli*, *P. aeruginosa*, *K. pneumoniae*, and *A. baumannii* and are shown in Table 1. All the EPIs, except for **3**, exhibited intrinsic antibacterial activities higher than the tested concentrations. Moreover, **3** displayed weak–moderate antibacterial activity in *E. coli*, *K. pneumoniae*, and *P. aeruginosa* (MIC = 32, 64, and 128 µg/mL, respectively).

Compounds **6** and **7** showed strong antibacterial activity against *E. coli* and *A. baumannii* (MIC = 0.5 and 1 µg/mL for **6** and **7**, respectively); however, they exhibited weaker antibacterial activity with 8–16-fold higher MIC values against *P. aeruginosa* and *K. pneumoniae*. Weak intrinsic antibacterial activity of **8** was observed in all the tested strains. The NBTIs were also tested against *E. coli* D22, which has increased membrane permeability compared to the wild-type *E. coli* and *E. coli* N43 strain, which lacks the AcrA component of a major multidrug resistance pump. The intrinsic antibacterial activities in both strains were significantly lower than in the wild-type *E. coli*.

Additionally, the three ACIs were evaluated via the checkerboard assay against *E. coli*, *P. aeruginosa*, *K. pneumoniae*, and *A. baumannii* (Table 1). Compound **10** exhibited relatively good antibacterial activity against all the tested strains (MIC ≤ 2 µg/mL in all the strains), whereas **9** and **11** showed moderate inhibition of *E. coli*, *P. aeruginosa*, and *A. baumannii* and weaker activity against *K. pneumoniae*.

### 2.2. EPI Potentiation of NBTI Antibacterial Activity in Various Gram-Negative Bacteria

To assess whether any EPIs potentiated the activity of NBTIs against different Gram-negative bacteria, the MICs of the three investigated NBTIs were determined for *E. coli*, *P. aeruginosa*, *K. pneumoniae*, and *A. baumannii* in the presence and absence of different concentrations of EPIs.

In the presence of PAβN, **1**, there was a significant improvement in the antibacterial activity of the NBTIs. In *E. coli* and *K. pneumoniae*, a PAβN concentration of 4–8 µg/mL resulted in an 8-fold improvement in the MIC for compounds **6** and **7** (Figures S1 and S2). In *P. aeruginosa*, which is known as a problematic Gram-negative bacterium, an 8-fold improvement was achieved with a PAβN concentration of 16 µg/mL (Table 2) for both compounds, **6** and **7**. Exact values for **8** could not be determined due to the weak antibacterial activity of **8** alone (MIC > 128 µg/mL). For *P. aeruginosa* and *K. pneumonia*, however, at least an 8-fold reduction in MIC was observed with the addition of 32 µg/mL of PAβN. For the combination of **8** and PAβN, no correlation was observed in *E. coli* and *A. baumannii*, and the MIC could not be determined as the turbidity readout did not yield the expected linear increase in response to the increased concentration of each compound (Figure S3). No significant effects of **2**, **3**, **4**, and **5** were observed regarding the NBTIs (Figures S4–S12) in any bacterial species tested.

### 2.3. ACI and NBTI Checkerboard Assay Results

The effect of the combination of **6** and an ACI on the antibacterial potency in comparison to their individual activities was determined for *E. coli*, *P. aeruginosa*, *K. pneumoniae*, and *A. baumannii* (Table 3, Figures S13–S15).

The MIC values of the compounds administered in combination generally decreased 2–4-fold compared to the compounds administered individually (Table 3). The calculated fractional inhibitory concentration (FIC) indices revealed an additive effect for all the combinations of **6** with the three ACIs in all the bacteria (Table 4). No significant synergistic effect was observed in any of the assayed combinations. FIC is a measure used to assess the interaction between two antimicrobial agents. An FIC index below 0.5 signifies synergistic interaction between the two tested antibacterials, values of the index between 0.5 and 4 are considered as an additive effect or indifference, and an index value above 4 signifies an antagonistic interaction between the two tested drugs.

### 2.4. Inhibition of A. baumannii Biofilm Formation and Elimination of Mature Biofilms

**Figure 3:**
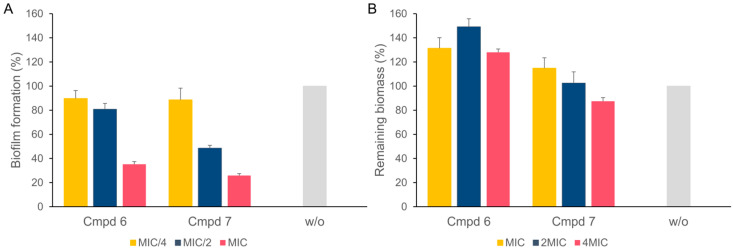
Inhibition of *A. baumannii* biofilm formation (**A**) and mature (24 h old) *A. baumannii* biofilm disintegration (**B**) with compounds **6** and **7** at different concentrations. Treatment with DMSO (w/o (without the compound), grey) was used as negative control.

Compounds **6** and **7** showed a dose-dependent reduction in the *A. baumannii* biofilms, achieving 65% and 75% inhibition at the MIC concentration, respectively (Figure 3A). Compound **6** applied to 24 h old biofilms slightly stimulated the biofilm growth, while **7** had no significant effect on mature biofilms at concentrations of up to 4 × MIC (Figure 3B).

**Figure 4:**
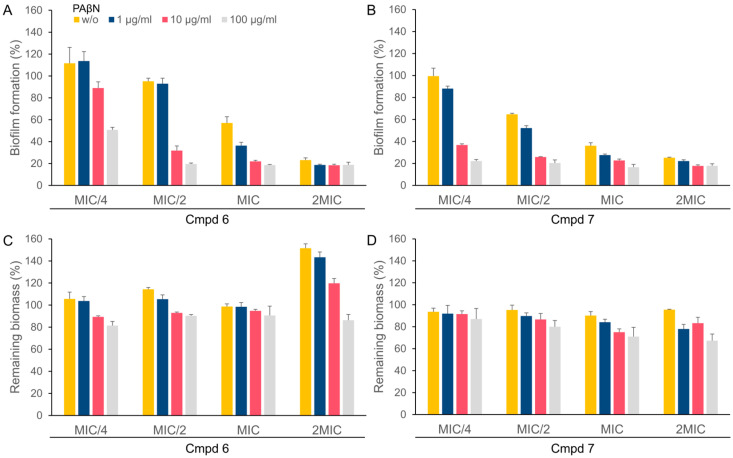
Inhibition of biofilm formation (**A**,**B**) and biofilm disintegration (**C**,**D**) with compounds **6** and **7** at concentrations equal to MIC/4, MIC/2, MIC, and 2MIC (MIC1 = 0.125 µg/mL; MIC2 = 1 µg/mL), alone (w/o), and with increasing concentrations of PAβN (1 µg/mL, 10 µg/mL, and 100 µg/mL).

Since PAβN significantly lowered the MIC of the NBTIs, we investigated its effect on biofilm formation (Figure 4A,B) and mature biofilm disruption (Figure 4C,D) when used in combination with compounds **6** and **7** at different concentrations.

PAβN alone did not inhibit the biofilm formation at any of the concentrations tested (Figure S16). However, when PAβN was used in combination with compounds **6** and **7**, a significant increase in the inhibition of biofilm formation was observed. In the case of compound **6**, additional inhibition of biofilm formation occurred in combination with PAβN in all the combinations except at concentrations of compound **6** equal to MIC/4 and MIC/2 and 1 µg/mL PAβN, where the effect was similar to that of compound **6** alone (Figure 4A). All the combinations of compound **7** with PAβN resulted in additional inhibition of biofilm formation compared to compound **7** alone (Figure 4B). Approximately an 80% reduction in biofilm formation was achieved when the bacteria were exposed to the concentration of compounds **6** and **7** at 2 × MIC or when the compounds were combined with 100 µg/mL PAβN.

In combination with PAβN, a slight dose-dependent mature biofilm disintegration was observed for both compounds (Figure 4C,D). In combination with 100 µg/mL PAβN, a reduction in the biofilm mass of approximately 20 to 25% was achieved at each concentration of the compounds. PAβN alone showed no significant effect against mature biofilms (Figure S16).

## 3. Discussion

This study focused on discovering novel ways of enhancing the antimicrobial activity of a series of NBTIs against Gram-negative bacteria by assessing various different approaches, including combining them with different EPIs and ACIs. The difference in the intrinsic antibacterial activity of the NBTIs between *E. coli* D22 and the wild-type *E. coli* showed that our compounds struggle with cell permeability, especially compound **7**, whose MIC for *E. coli* D22 is much lower compared to the MIC for the wild-type *E. coli*. The MIC values of the NBTIs against the *E. coli* N43 strains were significantly lower for all three tested compounds, indicating that the compounds are excellent substrates for *E. coli* efflux pumps; therefore, combining them with efflux pump inhibitors would be a sensible strategy for improving the antibacterial activity of these compounds. To verify that the tested EPIs were not acting as growth inhibitors, the MIC values for **1**–**5** were determined. Only **3** displayed weak–moderate antibacterial activity in *E. coli*, *K. pneumoniae*, and *P. aeruginosa*.

The EPIs used in this paper were selected because we wished to explore a range of differently acting commercially available EPIs that have been reported in the literature. PAβN was the only EPI where there was a significant improvement in the antibacterial activity of the NBTIs. As indicated in Table 2, an increase in the concentration of PAβN progressively lowers the MIC of the antibacterials. The MPC_8_ values for **6** and **7** in *E. coli* and *K. pneumoniae* were achieved at the PAβN concentrations of 4–8 µg/mL and 16 µg/mL in *P. aeruginosa* (Table 2). The exact values for **8** could not be determined due to the weak antibacterial activity of **8** alone (MIC > 128 µg/mL); however, at least an 8-fold reduction in MIC was observed with the addition of 32 µg/mL PAβN in *P. aeruginosa* and *K. pneumoniae.* NMP showed no significant effects in combination with the NBTIs in any bacteria (Figures S4–S6). The other tested EPIs, CCCP, reserpine, and berberine, in combination with **6** and **7**, did not show any significant improvement in antibacterial activity in *E. coli*, *P. aeruginosa*, and *K. pneumoniae* (Figures S7–S12). This was expected due to a different mechanism of efflux pump inhibition by 3, and the fact that reserpine and berberine target the efflux pumps of Gram-positive bacteria. The efflux pump inhibitors were not used against Gram-positive bacteria since the Gram-positive bacteria (e.g., *S. aureus* and MRSA) were found to be more susceptible to the NBTIs tested (with MICs as low as 0.004 µg/mL) [37,39,40]. In addition to their potentiation of the antibacterial activity of NBTIs, an important aspect of the investigated EPIs is their toxicity. Toxicity can be measured in vivo and expressed as a minimum lethal dose (MLD), i.e., the minimum dose that results in lethality in ≥66% of the animals tested [45]. As mentioned in the Introduction, PAβN exhibits a moderate toxic effect in a mouse model with an MLD < 25 mg/kg [45]. In our case, a PAβN concentration of 16 µg/mL resulted in an 8–32-fold improvement in MIC for the tested NBTIs, suggesting that safety issues might arise at such PAβN concentrations in in vivo studies (Table 2). However, there are analogues of PAβN with better safety profiles, so we plan to continue our future studies by using them in combination with selected NBTIs. The combination approach, as described in this paper, shows improved efficacy regarding the antibacterials and could potentially be utilized for future treatment of Gram-negative bacterial infections. As mentioned in the Introduction, however, the clinical validation of such an approach is still needed. The discovery and development of novel less-toxic EPIs might pave the way for such an approach to become reality.

To explore whether the coadministration of NBTIs and ACIs is a valid strategy for improving the antibacterial potency of the two drug classes, the activity of compound **6** against various Gram-negative bacteria in the presence of different concentrations of **9**, **10**, and **11** was investigated. No synergy was observed in any combination, only additive effects (Table 4), meaning that the drugs show a combined effect similar to what would be expected if they were acting independently. The combination is neither particularly beneficial nor harmful. The coadministration of the two topoisomerase inhibitor classes with different modes of action could have resulted in synergism by increasing the overall target inhibition. An additive effect might have occurred rather than synergistic action because both classes of compounds act on the same target enzyme instead of inhibiting multiple targets at the same time. It would be interesting to combine NBTIs with antibacterials that disrupt other vital cellular processes to see if this results in synergy. Both synergy and additive effects are welcome outcomes. A synergistic effect is beneficial for the treatment of difficult-to-treat bacterial infections, whereas an additive effect may be applied to broadening antimicrobial coverage and avoiding toxicity issues by lowering the administered concentrations of both antibacterials.

Moreover, **6** and **7** alone inhibit *A. baumannii* biofilm formation in a dose-dependent manner; **6** achieved 65% inhibition and **7** achieved 75% inhibition at the concentration of the compounds equal to the MIC. Combining them with PAβN potentiates biofilm inhibition as compared to NBTIs alone, making them useful for the prevention of biofilms. This inhibition is especially relevant for *A. baumannii*, a pathogen known to cause persistent and difficult-to-treat infections due to its biofilm-forming ability. NBTIs alone or in combination with PAβN, however, are not able to significantly disrupt mature biofilms. While PAβN slightly improved mature biofilm disruption, the effect was relatively modest (around 20–25% biofilm reduction), and the compounds remained largely ineffective against mature biofilms and would therefore be ineffective for the treatment of chronic biofilm-associated infections.

## 4. Materials and Methods

### 4.1. Bacterial Strains

Reference strains of *E. coli*, *P. aeruginosa*, *K. pneumoniae*, and *A. baumannii* were obtained from different libraries, *E. coli* from ATCC (*E. coli* (ATCC 25922)), *P. aeruginosa* from DSM (*P. aeruginosa* RDK 184 (DSM 939)), *K. pneumoniae* from RDK 070A (ATCC 51503), and *A. baumannii* from 8C6 (GES-14).

### 4.2. Chemicals and Media

PAβN, NMP, CCCP, reserpine, and berberine were purchased from Sigma-Aldrich (Steinheim, Germany). NBTIs and ACIs were synthesized in house and have been previously characterized for purity [36,38,40,46,47,48].

### 4.3. Determination of MIC—Susceptibility Testing

The minimum inhibitory concentrations (MICs) were determined by broth micro-dilution method in 96-well plate format according to the guidelines of Clinical and Laboratory Standards Institute and recommendations of European Committee on Antimicrobial Susceptibility Testing [49,50]. Bacterial suspension of the specific bacterial strain (*E. coli*, *P. aeruginosa*, *K. pneumoniae*, and *A. baumannii*) equivalent to the 0.5 McFarland turbidity standard was diluted with cation-adapted Mueller–Hinton broth with TES (Thermo Fisher Scientific, Waltham, MA, USA) to obtain a final inoculum of 10^5^ CFU/mL. The compounds (NBTIs, ACIs, and EPIs), dissolved in DMSO, and the inoculum were mixed together and incubated at 35 °C for 20 h. The assay concentrations of the tested compounds ranged from 256 to 0.004 µg/mL. After incubation, the MIC values were determined by visual inspection as the lowest dilution of the compounds showed no turbidity. Tetracycline was used as a positive control on each test plate.

### 4.4. Determination of MPCn

Minimum potentiation concentration (MPC) is defined as the lowest concentration of an EPI that causes a n-fold reduction in the minimum inhibitory concentration MIC of an antibiotic (expressed as MPC_n_ for an n-fold reduction) [23]. To determine MPC_8_ values, a checkerboard assay was designed with single-agent concentrations of NBTI and EPI to determine the MIC and combinations of different concentrations of NBTI and EPI. We added serial dilutions of EPI to columns 1–10; the highest concentration was in column 1 and the lowest in column 10. No EPI was added to column 11. Serial dilutions of an NBTI were added to rows A–H; the highest concentration was in row A and the lowest in row G. No NBTI was added to row H. Column 12 contained dilutions of tetracycline as a control. We first determined MIC_NBTI_ in the presence of different concentrations of PAβN (Table 2) and then chose MIC_EPI_ required to reduce the MIC_NBTI_ at least 8-fold (MPC_8_). Indeed, 8-fold greater reduction in MIC_NBTI_ values after addition of EPI was considered significant. The assay concentrations of the EPIs used in the checkerboard assay ranged from 256 to 0.5 µg/mL for **2**, from 128 to 0.25 µg/mL for **1**, **3**, and **4**, and from 64 to 0.125 µg/mL in **5**. The assay concentrations of NBTIs depended on the bacterial strains used in the assay. Assay concentrations of **6** ranged from 16 to 0.25 µg/mL in *P. aeruginosa*, from 4 to 0.063 µg/mL in *K. pneumoniae*, and from 1 to 0.016 µg/mL in *E. coli* or *A. baumannii*. Assay concentrations of **7** ranged from 32/16 to 0.5/0.25 µg/mL in *P. aeruginosa*, from 16/8 to 0.25/0.125 µg/mL in *K. pneumoniae*, from 2 to 0.031 µg/mL in *E. coli*, and from 2/1 to 0.031/0.016 µg/mL in *A. baumannii*. Assay concentrations of **8** ranged from 128 to 2 µg/mL in all tested bacterial strains.

### 4.5. Checkerboard Assay and the Determination of FIC Index

Fractional inhibitory concentration (FIC) is defined as the sum of the FICs of each drug tested (${\mathrm{FIC}}_{A},\;{\mathrm{FIC}}_{B}$), where the FIC for each drug is determined by dividing the MIC of each drug in combination ($C_{A},\;C_{B}$) by the MIC of each drug when used alone (${\mathrm{MIC}}_{A},\;{\mathrm{MIC}}_{B}$) (Equation (1)):

(1) $$\mathrm{FIC}={\mathrm{FIC}}_{A}+{\mathrm{FIC}}_{B}=\frac{C_{A}}{{\mathrm{MIC}}_{A}}+\frac{C_{B}}{{\mathrm{MIC}}_{B}}$$

Synergy is assumed when the value of the FIC index is <0.5 and antagonism when FIC > 4. In synergy, the combination of compounds increases the inhibitory activity (decrease in MIC) of one or both compounds compared to just the compounds alone, while in antagonism more compound would be required in order to produce the same effect as the compounds alone. When the combination of compounds results in an FIC value of 0.5–4, the combination has no increase in inhibitory activity or a slight increase in inhibitory activity from the additive effect of both compounds combined. A checkboard assay, similar to the MPC_8_ determination assay, was designed. NBTIs were added to columns 2–11; column 2 contained the lowest concentration, followed by a 2-fold increase in concertation in each consecutive column. No NBTI was added to the first column; therefore, intrinsic antibacterial activity of the ACIs could be determined. ACIs were added to rows A–G; row A contained the highest concentration of ACI, followed by a 2-fold decrease in concertation in each consecutive row. No ACI was added to row H; therefore, we could determine the intrinsic antibacterial activity of the NBTI. Column 12 contained 2-fold dilutions of tetracycline as a control. Assay concentrations ranged from 16 to 0.031 µg/mL for **6**, from 64 to 1 µg/mL for **9** and **11**, and from 16 to 0.031 µg/mL for **10** in all tested combinations.

### 4.6. Biofilm Formation Inhibition Assay

Biofilm quantification assays were performed in 96-well microtiter plates using a crystal violet (CV) method to stain adherent cells [51]. Overnight cultures of *A. baumanii* ATCC 19606 were diluted to 5 × 10^7^ cells mL^−1^ in brain heart infusion media (BHI), and 100 µL was added to the wells without treatment (negative control) or in the presence of the compounds. Biofilms formed for 24 h at 37 °C were washed and adherent cells stained with 0.1% CV (*v*/*v*). Assays were performed in six wells and repeated two times.

### 4.7. Mature Biofilm Disruption Assay

Overnight cultures of *A. baumannii* were diluted to 5 × 10^7^ cells mL^−1^ in BHI and 100 μL was added to the wells in 96-well microtiter plates and incubated for 24 h at 37 °C. After removal of the culture from the wells and two washing steps with PBS, adherent cells were exposed to different concentrations of the compounds for additional 24 h and the biofilm biomass was quantified using CV as described above. The wells without treatment served as a control. Assays were performed in six wells and repeated two times.

### 4.8. Potentiation of Compounds’ Antibiofilm Activity with Efflux Pump Inhibitors

To investigate whether PAβN can potentiate antibiofilm activity of the compounds, compounds **6** and **7** at concentrations MIC/4, MIC/2, MIC, and 2 × MIC were combined with increasing concentrations of PAβN (1, 10, and 100 μg/mL), and biofilm formation inhibition assay and mature biofilm disruption assay were performed as described above.

## 5. Conclusions

With the steady increase in antimicrobial resistance, multidrug-resistant bacteria are becoming increasingly difficult to fight. There are fewer and fewer effective antimicrobials, so new approaches are needed. In this study, we presented a few such approaches: coadministration of an antimicrobial agent (NBTI) and an efflux pump inhibitor (EPI), coadministration of two antibacterials with distinct modes of action (NBTI and ACI), as well as inhibition of biofilm formation and mature biofilm disruption. We investigated a combination of three NBTIs with different EPIs against Gram-negative bacteria belonging to ESKAPE pathogens, as well as a combination of an NBTI with three different ACIs. Among the EPIs tested, PAβN significantly improved the antibacterial activity of NBTIs against Gram-negative bacteria (more than 8-fold). In contrast, the other EPIs tested (e.g., NMP, CCCP, reserpine, and berberine) did not improve the antibacterial activity of the NBTIs against selected Gram-negative bacteria. The investigated combinations of NBTI with ACIs resulted in an additive effect or indifference but did not produce a synergistic antibacterial effect. NBTIs alone and in combination with PAβN inhibited biofilm formation but were less effective in inhibiting mature biofilms. Our results demonstrate for the first time that the combination of NBTIs with EPI could improve the effectiveness of NBTIs and enable their clinical use, especially when the NBTIs are known efflux pump substrates. However, it is vital to develop new EPIs with improved safety profiles in the future. Combining NBTIs with ACIs did not show a synergistic effect, yet the combination itself might prove to be beneficial, especially in the therapy of resistant strains. Inhibition of biofilm formation and mature biofilm disruption are strategies aimed at reducing the virulence of aggregated bacteria known to cause chronic infections. Although our compounds were not successful in disrupting mature biofilms, they strongly inhibited biofilm formation. Overall, the results of this study will undoubtedly help to pave the way to developing more effective treatments for Gram-negative bacterial infections in the future.

## Figures and Tables

**Table 1:** Antibacterial activity of NBTIs and EPIs against various Gram-negative “ESKAPE” pathogens.

Bacterial Strain	MIC (µg/mL)										
	EPI					NBTI			ACI		
	1	2	3	4	5	6	7	8	9	10	11
*E. coli* (ATCC 25922)	>128	>256	32	>128	>64	0.5–1	2	128	8	1	8
*E. coli* D22 ^a^	ND	ND	ND	ND	ND	0.25	0.016	128	ND	ND	ND
*E. coli* N43 (CGSC# 5583) ^b^	ND	ND	ND	ND	ND	0.008	<0.008	0.031	ND	ND	ND
*P. aeruginosa* RDK 184	>128	>256	128	>128	>64	16	16	>128	16	1	8
*K. pneumoniae* RDK 070A	>128	>256	64	>128	>64	2	8	>128	16–32	2	16
*A. baumannii* 8C6 (GES-14)	>128	>256	ND	ND	ND	0.125–0.5	0.5–1	>128	8–16	1	4

^a^ With a mutation in the lpxC gene that increases membrane permeability. ^b^ With AcrA knock-out (cell membrane efflux pump). ND: not determined.

**Table 2:** Antibacterial activity of NBTIs in combination with efflux pump inhibitor PAβN.

Bacterial Strain	Cmpd	MIC of NBTI in the Presence of PAβN (µg/mL)										MPC_8_ ^b^ (µg/mL)
		PAβN ^a^ Concentration (µg/mL)										
		0	0.5	1	2	4	8	16	32	64	128	
*E. coli*	**6**	1	0.5	0.5	0.25	0.125	0.063	0.031	0.016	0.016	0.016	4
	**7**	2	2	2	1	0.5	0.25	0.063	0.031	0.031	0.031	8
*P. aeruginosa*	**6**	16	8	8	8	8	8	1	0.25	0.063	0.031	16
	**7**	16	8	8	8	8	8	2	0.5	0.25	0.125	16
	**8**	>128	>128	>128	>128	>128	>128	>128	8	2	2	16–32
*K. pneumoniae*	**6**	2	2	2	2	1	0.125	0.063	0.031	0.031	0.016	8
	**7**	8	8	8	8	2	1	0.25	0.125	0.063	0.031	8
	**8**	>128	>128	>128	>128	>128	>128	>128	2	2	2	16–32
*A. baumannii*	**6**	0.25	0.063	0.063	0.031	0.031	0.031	0.016	0.016	0.016	0.016	2
	**7**	0.5	0.5	0.5	0.5	0.25	0.25	0.125	0.063	0.031	0.031	32

MIC values of the antibacterials alone are shown in the third column, where PAβN concentration equals 0. All MPCs were determined by comparing the MICs of the antibacterials alone with the MICs of the antibacterials in combination with PAβN. ^a^ PAβN: efflux pump inhibitor Phe-Arg-naphthylamide. ^b^ MPC_8_: minimum concentration of efflux pump inhibitor in µg/mL required to reduce the MIC of NBTI at least 8-fold.

**Table 3:** MIC values of individual compounds or in combination.

Bacterial Strain	MIC (µg/mL)									
	NBTI				ACI					
	6				9		10		11	
	I ^a^	C ^b, 9^	C ^b, 10^	C ^b, 11^	I ^a^	C ^b, 6^	I ^a^	C ^b, 6^	I ^a^	C ^b, 6^
*E. coli*	0.5	0.25	0.25	0.125	8	4	1	0.5	8	4
*P. aeruginosa*	16	8	8	8	16	2	1	0.5	8	2
*K. pneumoniae*	2–4	2	2	1	16–32	8	2	1	16	8
*A. baumannii*	0.125	0.063	0.063	0.063	16	4	1	0.25	4	4

^a^ Intrinsic antibacterial activity, i.e., MIC when the compound is administered individually (I). ^b^ MIC when the compound is administered in combination (C). ^9^ MIC in combination with **9**. ^10^ MIC in combination with **10**. ^11^ MIC in combination with **11**. ^6^ MIC in combination with compound **6**.

**Table 4:** FIC index values of compound **6** in combination with various ACIs.

Bacterial Strain	FIC		
	C ^b, 9^	C ^b, 10^	C ^b, 11^
*E. coli*	1.000	1.000	0.750
*P. aeruginosa*	0.625	1.000	0.750
*K. pneumoniae*	0.750	1.000	0.750
*A. baumannii*	0.750	0.750	1.500

^b^ MIC when the compound is administered in combination (C). ^9^ MIC in combination with **9**. ^10^ MIC in combination with **10**. ^11^ MIC in combination with **11**.

## Data Availability

The original contributions presented in the study are included in the article/Supplementary Materials, further inquiries can be directed to the corresponding author.

## Supplementary Materials

The following supporting information can be downloaded at https://www.mdpi.com/article/10.3390/antibiotics13111081/s1, Figure S1: Checkerboard assay of PAβN (**1**) and **6** in different Gram-negative bacterial strains; Figure S2: Checkerboard assay of PAβN (**1**) and **7** in different Gram-negative bacterial strains; Figure S3: Checkerboard assay of PAβN (**1**) and **8** in different Gram-negative bacterial strains; Figure S4: Checkerboard assay of NMP (**2**) and **6** in different Gram-negative bacterial strains; Figure S5: Checkerboard assay of NMP (**2**) and **7** in different Gram-negative bacterial strains; Figure S6: Checkerboard assay of NMP (**2**) and **8** in different Gram-negative bacterial strains; Figure S7: Checkerboard assay of CCCP (**3**) and **6** in different Gram-negative bacterial strains; Figure S8: Checkerboard assay of CCCP (**3**) and **7** in different Gram-negative bacterial strains; Figure S9: Checkerboard assay of reserpine (**4**) and **6** in different Gram-negative bacterial strains; Figure S10: Checkerboard assay of reserpine (**4**) and **7** in different Gram-negative bacterial strains; Figure S11: Checkerboard assay of barberine (**5**) and **6** in different Gram-negative bacterial strains; Figure S12: Checkerboard assay of barberine (**5**) and **7** in different Gram-negative bacterial strains; Figure S13: Checkerboard assay **6** and **9** in different Gram-negative bacterial strains; Figure S14: Checkerboard assay **6** and **10** in different Gram-negative bacterial strains; Figure S15: Checkerboard assay **6** and **11** in different Gram-negative bacterial strains; Figure S16: Inhibition of *A. baumannii* biofilm formation (A) and biofilm disintegration (B) with PAβN at different concentrations.
