# Supplementary material for: Enhancing Antibacterial Efficacy: Combining Novel Bacterial Topoisomerase Inhibitors with Efflux Pump Inhibitors and Other Agents Against Gram-Negative Bacteria
Source: Antibiotics (Basel). 2024 Nov 13;13(11):1081. doi: 10.3390/antibiotics13111081 (PMC11591509; doi:10.3390/antibiotics13111081)

Supplementary Information

Enhancing Antibacterial Efficacy: Combining Novel Bacterial Topoisomerase Inhibitors with Efflux Pump Inhibitors and Other Agents Against Gram-Negative Bacteria

Maša Zorman [1,2], Maja Kokot [1,2], Irena Zdovc [3], Lidija Senerovic [4], Mina Mandic [4], Nace Zidar [2], Andrej Emanuel Cotman [2], Martina Durcik [2], Lucija Peterlin Mašič [2], Nikola Minovski [1], Marko Anderluh [2] and Martina Hrast Rambaher [2,*]

[1] Theory Department, Laboratory for Cheminformatics, National Institute of Chemistry, Hajdrihova 19, 1001 Ljubljana, Slovenia
[2] Department of Pharmaceutical Chemistry, Faculty of Pharmacy, University of Ljubljana, Aškerčeva Cesta 7, 1000 Ljubljana, Slovenia
[3] Institute of Microbiology and Parasitology, Veterinary Faculty, University of Ljubljana, Gerbičeva 60, 1000 Ljubljana, Slovenia
[4] Laboratory for Microbial Molecular Genetics and Ecology, Institute of Molecular Genetics and Genetic Engineering, University of Belgrade, Vojvode Stepe 444a, 11 042 Belgrade, Serbia

NBTI and EPI checkerboard assay

Grey color represents the growth of bacteria in all Figures.

Figure S1: Checkerboard assay of PAβN (1) and **6** in different Gram-negative bacterial strains.

E. coli

		1	2	3	4	5	6	7	8	9	10	11	12
	EPI → NBTI ↓	128	64	32	16	8	4	2	1	0.5	0.25	0	T
A	1												
B	0.5												
C	0.25												
D	0.125												
E	0.062												
F	0.031												
G	0.016												
H	0												

P. aeruginosa

		1	2	3	4	5	6	7	8	9	10	11	12
	EPI → NBTI ↓	128	64	32	16	8	4	2	1	0.5	0.25	0	T
A	16												
B	8												
C	4												
D	2												
E	1												
F	0.5												
G	0.25												
H	0												

K. pneumoniae

		1	2	3	4	5	6	7	8	9	10	11	12
	EPI → NBTI ↓	128	64	32	16	8	4	2	1	0.5	0.25	0	T
A	4												
B	2												
C	1												
D	0.5												
E	0.25												
F	0.125												
G	0.063												
H	0												

A. baumannii

		1	2	3	4	5	6	7	8	9	10	11	12
	EPI → NBTI ↓	128	64	32	16	8	4	2	1	0.5	0.25	0	T
A	1												
B	0.5												
C	0.25												
D	0.125												
E	0.062												
F	0.031												
G	0.016												
H	0												

Figure S2: Checkerboard assay of PAβN (1) and **7** in different Gram-negative bacterial strains.

E. coli

		1	2	3	4	5	6	7	8	9	10		11	12
	EPI → / NBTI ↓	128	64	32	16	8	4	2	1	0.5	0.25		0	T
A	2													
B	1													
C	0.5													
D	0.25													
E	0.125													
F	0.063													
G	0.031													
H	0													

P. aeruginosa

		1	2	3	4	5	6	7	8	9	10	11	12
	EPI → / NBTI ↓	128	64	32	16	8	4	2	1	0.5	0.25	0	T
A	16												
B	8												
C	4												
D	2												
E	1												
F	0.5												
G	0.25												
H	0												

K. pneumoniae

		1	2	3	4	5	6	7	8	9	10	11	12
	EPI → / NBTI ↓	128	64	32	16	8	4	2	1	0.5	0.25	0	T
A	8												
B	4												
C	2												
D	1												
E	0.5												
F	0.25												
G	0.125												
H	0												

A. baumannii

		1	2	3	4	5	6	7	8	9	10	11	12
	EPI → / NBTI ↓	128	64	32	16	8	4	2	1	0.5	0.25	0	T
A	1												
B	0.5												
C	0.25												
D	0.125												
E	0.063												
F	0.031												
G	0.016												
H	0												

Figure S3: Checkerboard assay of PAβN (1) and **8** in different Gram-negative bacterial strains.

E. coli

		1	2	3	4	5	6	7	8	9	10	11	12
	EPI → / NBTI ↓	128	64	32	16	8	4	2	1	0.5	0.25	0	T
A	128												
B	64												
C	32												
D	16												
E	8												
F	4												
G	2												
H	0												

P. aeruginosa

		1	2	3	4	5	6	7	8	9	10	11	12
	EPI → / NBTI ↓	128	64	32	16	8	4	2	1	0.5	0.25	0	T
A	128												
B	64												
C	32												
D	16												
E	8												
F	4												
G	2												
H	0												

K. pneumoniae

		1	2	3	4	5	6	7	8	9	10	11	12
	EPI → / NBTI ↓	128	64	32	16	8	4	2	1	0.5	0.25	0	T
A	128												
B	64												
C	32												
D	16												
E	8												
F	4												
G	2												
H	0												

A. baumannii

		1	2	3	4	5	6	7	8	9	10	11	12
	EPI → / NBTI ↓	128	64	32	16	8	4	2	1	0.5	0.25	0	T
A	128												
B	64												
C	32												
D	16												
E	8												
F	4												
G	2												
H	0												

Figure S4: Checkerboard assay of NMP (2) and **6** in different Gram-negative bacterial strains.

E. coli

		1	2	3	4	5	6	7	8	9	10	11	12
	EPI →<br>NBTI ↓	256	128	64	32	16	8	4	2	1	0.5	0	T
A	1												
B	0,5												
C	0.25												
D	0.125												
E	0.062												
F	0.31												
G	0.016												
H	0												

P. aeruginosa

		1	2	3	4	5	6	7	8	9	10	11	12
	EPI →<br>NBTI ↓	256	128	64	32	16	8	4	2	1	0.5	0	T
A	16												
B	8												
C	4												
D	2												
E	1												
F	0.5												
G	0.25												
H	0												

K. pneumoniae

		1	2	3	4	5	6	7	8	9	10	11	12
	EPI →<br>NBTI ↓	256	128	64	32	16	8	4	2	1	0.5	0	T
A	4												
B	2												
C	1												
D	0.5												
E	0.25												
F	0.125												
G	0.062												
H	0												

A. baumannii

		1	2	3	4	5	6	7	8	9	10	11	12
	EPI →<br>NBTI ↓	256	128	64	32	16	8	4	2	1	0.5	0	T
A	1												
B	0.5												
C	0.25												
D	0.125												
E	0.062												
F	0.031												
G	0.016												
H	0												

Figure S5: Checkerboard assay of NMP (2) and **7** in different Gram-negative bacterial strains.

E. coli

		1	2	3	4	5	6	7	8	9	10	11	12
	EPI →<br>NBTI ↓	256	128	64	32	16	8	4	2	1	0.5	0	T
A	2												
B	1												
C	0.5												
D	0.25												
E	0.125												
F	0.062												
G	0.031												
H	0												

P. aeruginosa

		1	2	3	4	5	6	7	8	9	10	11	12
	EPI →<br>NBTI ↓	256	128	64	32	16	8	4	2	1	0.5	0	T
A	32												
B	16												
C	8												
D	4												
E	2												
F	1												
G	0.5												
H	0												

K. pneumoniae

		1	2	3	4	5	6	7	8	9	10	11	12
	EPI →<br>NBTI ↓	256	128	64	32	16	8	4	2	1	0.5	0	T
A	16												
B	8												
C	4												
D	2												
E	1												
F	0.5												
G	0.25												
H	0												

A. baumannii

		1	2	3	4	5	6	7	8	9	10	11	12
	EPI →<br>NBTI ↓	256	128	64	32	16	8	4	2	1	0.5	0	T
A	2												
B	1												
C	0.5												
D	0.25												
E	0.125												
F	0.062												
G	0.031												
H	0												

Figure S6: Checkerboard assay of NMP (2) and **8** in different Gram-negative bacterial strains.

E. coli

		1	2	3	4	5	6	7	8	9	10	11	12
	EPI →<br>NBTI ↓	256	128	64	32	16	8	4	2	1	0.5	0	T
A	128												
B	64												
C	32												
D	16												
E	8												
F	4												
G	2												
H	0												

P. aeruginosa

		1	2	3	4	5	6	7	8	9	10	11	12
	EPI →<br>NBTI ↓	256	128	64	32	16	8	4	2	1	0.5	0	T
A	128												
B	64												
C	32												
D	16												
E	8												
F	4												
G	2												
H	0												

K. pneumoniae

		1	2	3	4	5	6	7	8	9	10	11	12
	EPI →<br>NBTI ↓	256	128	64	32	16	8	4	2	1	0.5	0	T
A	128												
B	64												
C	32												
D	16												
E	8												
F	4												
G	2												
H	0												

A. baumannii

		1	2	3	4	5	6	7	8	9	10	11	12
	EPI →<br>NBTI ↓	256	128	64	32	16	8	4	2	1	0.5	0	T
A	128												
B	64												
C	32												
D	16												
E	8												
F	4												
G	2												
H	0												

Figure S7: Checkerboard assay of CCCP (3) and **6** in different Gram-negative bacterial strains.

E. coli

		1	2	3	4	5	6	7	8	9	10	11	12
	EPI →<br>NBTI ↓	128	64	32	16	8	4	2	1	0.5	0.25	0	T
A	1												
B	0.5												
C	0.25												
D	0.125												
E	0.062												
F	0.031												
G	0.016												
H	0												

P. aeruginosa

		1	2	3	4	5	6	7	8	9	10	11	12
	EPI →<br>NBTI ↓	128	64	32	16	8	4	2	1	0.5	0.25	0	T
A	16												
B	8												
C	4												
D	2												
E	1												
F	0.5												
G	0.25												
H	0												

K. pneumoniae

		1	2	3	4	5	6	7	8	9	10	11	12
	EPI →<br>NBTI ↓	128	64	32	16	8	4	2	1	0.5	0.25	0	T
A	4												
B	2												
C	1												
D	0.5												
E	0.25												
F	0.125												
G	0.062												
H	0												

Figure S8: Checkerboard assay of CCCP (3) and **7** in different Gram-negative bacterial strains.

E. coli

		1	2	3	4	5	6	7	8	9	10	11	12
	EPI →<br>NBTI ↓	128	64	32	16	8	4	2	1	0.5	0.25	0	T
A	2												
B	1												
C	0.5												
D	0.25												
E	0.125												
F	0.062												
G	0.031												
H	0												

P. aeruginosa

		1	2	3	4	5	6	7	8	9	10	11	12
	EPI →<br>NBTI ↓	128	64	32	16	8	4	2	1	0.5	0.25	0	T
A	32												
B	16												
C	8												
D	4												
E	2												
F	1												
G	0.5												
H	0												

K. pneumoniae

		1	2	3	4	5	6	7	8	9	10	11	12
	EPI →<br>NBTI ↓	128	64	32	16	8	4	2	1	0.5	0.25	0	T
A	16												
B	8												
C	4												
D	2												
E	1												
F	0.5												
G	0.25												
H	0												

Figure S9: Checkerboard assay of reserpine (4) and **6** in different Gram-negative bacterial strains.

E. coli

		1	2	3	4	5	6	7	8	9	10	11	12
	EPI →<br>NBTI ↓	128	64	32	16	8	4	2	1	0.5	0.25	0	T
A	1												
B	0.5												
C	0.25												
D	0.125												
E	0.062												
F	0.031												
G	0.016												
H	0												

P. aeruginosa

		1	2	3	4	5	6	7	8	9	10	11	12
	EPI →<br>NBTI ↓	128	64	32	16	8	4	2	1	0.5	0.25	0	T
A	16												
B	8												
C	4												
D	2												
E	1												
F	0.5												
G	0.25												
H	0												

K. pneumoniae

		1	2	3	4	5	6	7	8	9	10	11	12
	EPI →<br>NBTI ↓	128	64	32	16	8	4	2	1	0.5	0.25	0	T
A	4												
B	2												
C	1												
D	0.5												
E	0.25												
F	0.125												
G	0.062												
H	0												

Figure S10: Checkerboard assay of reserpine (4) and **7** in different Gram-negative bacterial strains.

E. coli

		1	2	3	4	5	6	7	8	9	10	11	12
	EPI →<br>NBTI ↓	128	64	32	16	8	4	2	1	0.5	0.25	0	T
A	2												
B	1												
C	0.5												
D	0.25												
E	0.125												
F	0.062												
G	0.031												
H	0												

P. aeruginosa

		1	2	3	4	5	6	7	8	9	10	11	12
	EPI →<br>NBTI ↓	128	64	32	16	8	4	2	1	0.5	0.25	0	T
A	32												
B	16												
C	8												
D	4												
E	2												
F	1												
G	0.5												
H	0												

K. pneumoniae

		1	2	3	4	5	6	7	8	9	10	11	12
	EPI →<br>NBTI ↓	128	64	32	16	8	4	2	1	0.5	0.25	0	T
A	16												
B	8												
C	4												
D	2												
E	1												
F	0.5												
G	0.25												
H	0												

Figure S11: Checkerboard assay of barberine (5) and **6** in different Gram-negative bacterial strains.

E. coli

		1	2	3	4	5	6	7	8	9	10	11	12
	EPI →<br>NBTI ↓	64	32	16	8	4	2	1	0.5	0.25	0.125	0	T
A	1												
B	0.5												
C	0.25												
D	0.125												
E	0.062												
F	0.031												
G	0.016												
H	0												

P. aeruginosa

		1	2	3	4	5	6	7	8	9	10	11	12
	EPI →<br>NBTI ↓	64	32	16	8	4	2	1	0.5	0.25	0.125	0	T
A	16												
B	8												
C	4												
D	2												
E	1												
F	0.5												
G	0.25												
H	0												

K. pneumoniae

		1	2	3	4	5	6	7	8	9	10	11	12
	EPI →<br>NBTI ↓	64	32	16	8	4	2	1	0.5	0.25	0.125	0	T
A	4												
B	2												
C	1												
D	0.5												
E	0.25												
F	0.125												
G	0.062												
H	0												

Figure S12: Checkerboard assay of berberine (5) and **7** in different Gram-negative bacterial strains.

E. coli

		1	2	3	4	5	6	7	8	9	10	11	12
	EPI → NBTI ↓	64	32	16	8	4	2	1	0.5	0.25	0.125	0	T
A	2												
B	1												
C	0.5												
D	0.25												
E	0.125												
F	0.062												
G	0.031												
H	0												

P. aeruginosa

		1	2	3	4	5	6	7	8	9	10	11	12
	EPI → NBTI ↓	64	32	16	8	4	2	1	0.5	0.25	0.125	0	T
A	32												
B	16												
C	8												
D	4												
E	2												
F	1												
G	0.5												
H	0												

K. pneumoniae

		1	2	3	4	5	6	7	8	9	10	11	12
	EPI → NBTI ↓	64	32	16	8	4	2	1	0.5	0.25	0.125	0	T
A	16												
B	8												
C	4												
D	2												
E	1												
F	0.5												
G	0.25												
H	0												

All concentrations are in µg/mL.

NBTI and ACI checkerboard assay

Figure S13: Checkerboard assay **6** and **9** in different Gram-negative bacterial strains.

E. coli

		1	2	3	4	5	6	7	8	9	10	11	12
	NBTI → / ACI ↓	0	0.03	0.06	0.13	0.25	0.5	1	2	4	8	16	T
A	64												4
B	32												2
C	16												1
D	8												0.5
E	4												0.25
F	2												0.13
G	1												0
H	0												0

P. aeruginosa

		1	2	3	4	5	6	7	8	9	10	11	12
	NBTI → / ACI ↓	0	0.03	0.06	0.13	0.25	0.5	1	2	4	8	16	T
A	64												4
B	32												2
C	16												1
D	8												0.5
E	4												0.25
F	2												0.13
G	1												0
H	0												0

K. pneumoniae

		1	2	3	4	5	6	7	8	9	10	11	12
	NBTI → / ACI ↓	0	0.03	0.06	0.13	0.25	0.5	1	2	4	8	16	T
A	64												4
B	32												2
C	16												1
D	8												0.5
E	4												0.25
F	2												0.13
G	1												0
H	0												0

A. baumannii

		1	2	3	4	5	6	7	8	9	10	11	12
	NBTI → / ACI ↓	0	0.03	0.06	0.13	0.25	0.5	1	2	4	8	16	T
A	64												4
B	32												2
C	16												1
D	8												0.5
E	4												0.25
F	2												0.13
G	1												0
H	0												0

Figure S14: Checkerboard assay **6** and **10** in different Gram-negative bacterial strains.

E. coli

		1	2	3	4	5	6	7	8	9	10	11	12
	NBTI → / ACI ↓	0	0.03	0.06	0.13	0.25	0.5	1	2	4	8	16	T
A	16												4
B	8												2
C	4												1
D	2												0.5
E	1												0.25
F	0.5												0.13
G	0.25												0
H	0												0

P. aeruginosa

		1	2	3	4	5	6	7	8	9	10	11	12
	NBTI → / ACI ↓	0	0.03	0.06	0.13	0.25	0.5	1	2	4	8	16	T
A	16												4
B	8												2
C	4												1
D	2												0.5
E	1												0.25
F	0.5												0.13
G	0.25												0
H	0												0

K. pneumoniae

		1	2	3	4	5	6	7	8	9	10	11	12
	NBTI → / ACI ↓	0	0.03	0.06	0.13	0.25	0.5	1	2	4	8	16	T
A	16												4
B	8												2
C	4												1
D	2												0.5
E	1												0.25
F	0.5												0.13
G	0.25												0
H	0												0

A. baumannii

		1	2	3	4	5	6	7	8	9	10	11	12
	NBTI → / ACI ↓	0	0.03	0.06	0.13	0.25	0.5	1	2	4	8	16	T
A	16												4
B	8												2
C	4												1
D	2												0.5
E	1												0.25
F	0.5												0.13
G	0.25												0
H	0												0

Figure S15: Checkerboard assay **6** and **11** in different Gram-negative bacterial strains.

E. coli

		1	2	3	4	5	6	7	8	9	10	11	12
	NBTI → / ACI ↓	0	0.03	0.06	0.13	0.25	0.5	1	2	4	8	16	T
A	64												4
B	32												2
C	16												1
D	8												0.5
E	4												0.25
F	2												0.13
G	1												0
H	0												0

P. aeruginosa

		1	2	3	4	5	6	7	8	9	10	11	12
	NBTI → / ACI ↓	0	0.03	0.06	0.13	0.25	0.5	1	2	4	8	16	T
A	64												4
B	32												2
C	16												1
D	8												0.5
E	4												0.25
F	2												0.13
G	1												0
H	0												0

K. pneumoniae

		1	2	3	4	5	6	7	8	9	10	11	12
	NBTI → / ACI ↓	0	0.03	0.06	0.13	0.25	0.5	1	2	4	8	16	T
A	64												4
B	32												2
C	16												1
D	8												0.5
E	4												0.25
F	2												0.13
G	1												0
H	0												0

A. baumannii

		1	2	3	4	5	6	7	8	9	10	11	12
	NBTI → / ACI ↓	0	0.03	0.06	0.13	0.25	0.5	1	2	4	8	16	T
A	64												4
B	32												2
C	16												1
D	8												0.5
E	4												0.25
F	2												0.13
G	1												0
H	0												0

All concentrations are in µg/mL.

Inhibition of *A. baumannii* biofilm formation and mature biofilm disintegration

Figure S16: Inhibition of *A. baumannii* biofilm formation (A) and biofilm disintegration (B) with PAβN at different concentrations.

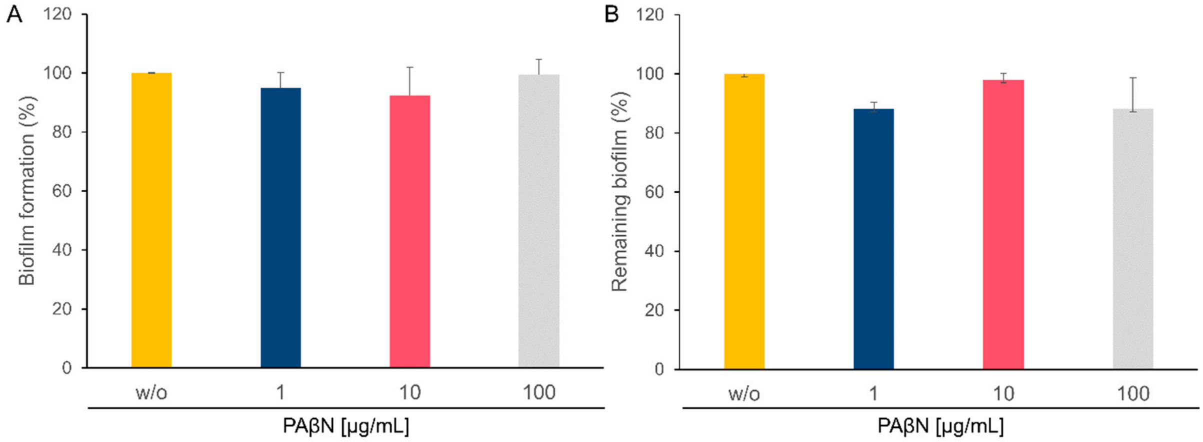

Supplement: Supplementary file 1 [file antibiotics-13-01081-s001.zip › antibiotics-3310491-supplementary.pdf]
